# Assessing psychological antecedents of vaccination in family health center visitors

**DOI:** 10.1017/S146342362610098X

**Published:** 2026-02-18

**Authors:** Cansu Özbaş, Merve Tokatlı Doğan, Fatma Nur Baran Aksakal

**Affiliations:** 1 Department of Public Health, Faculty of Medicine, Gazi University, Ankara, Türkiye; 2 Istanbul Provincial Health Directorate: Istanbul Il Saglik Mudurlugu, Türkiye

**Keywords:** 5C model, decision-making, public health, vaccination intentions, vaccine hesitancy

## Abstract

**Objectives:**

This study evaluates the psychological factors influencing vaccination attitudes and behaviors among individuals visiting family health centers. Using the 5C model, the study identifies key determinants affecting vaccination intention and hesitancy, providing insights into strategies to enhance vaccine acceptance.

**Methods:**

A cross-sectional study was conducted among 1712 adults aged 18 and over in Ankara, Turkey. Data were collected through face-to-face interviews, covering socio-demographic characteristics, vaccination attitudes, and internet usage. The Turkish version of the ‘Psychological Antecedents of Vaccination (5C) Scale’ was used to assess participants’ responses. Multivariate logistic regression analysis was performed to determine factors influencing vaccination intention.

**Results:**

Individuals whose income exceeds their expenses (OR: 1.532, 95% CI: 1.107–2.119), those who received the COVID-19 vaccine (OR: 2.362, 95% CI: 1.429–3.906), and those who are not active social media users (OR: 1.417, 95% CI: 1.096–1.833) were more likely to get vaccinated without hesitation. Higher **confidence** (OR: 1.268, 95% CI: 1.231–1.306) and **collective responsibility** (OR: 1.083, 95% CI: 1.046–1.122) scores were associated with increased willingness to vaccinate, while higher **calculation** scores (OR: 0.932, 95% CI: 0.899–0.965) were linked to hesitancy.

**Conclusions:**

The findings suggest that fostering **confidence and collective responsibility** is crucial for improving vaccine acceptance. Communication strategies should be tailored to reduce hesitancy among active social media users. Future research should explore the underlying risk factors contributing to vaccine hesitancy in different populations.

## Introduction

Immunization plays a crucial role in public health by providing widespread protection against infectious diseases and supporting societal well-being. However, vaccine hesitancy poses a significant barrier to achieving optimal vaccination coverage and ensuring herd immunity (Vigezzi *et al*., 2024). The World Health Organization (WHO) has identified vaccine hesitancy as one of the main reasons for lower vaccination rates, listing it among the top ten global public health threats, particularly in low – and middle-income countries. WHO defines vaccine hesitancy as a delay in acceptance or refusal of vaccines despite the availability of vaccination services. Vaccine hesitancy varies by time, place, and type of vaccine, making it a complex and context-specific issue (Aggarwal *et al*., 2024; Yang *et al*., 2024). Addressing vaccine hesitancy is crucial for the success of immunization programs, as understanding the factors influencing vaccination behavior is key to controlling infectious diseases (Betsch *et al*., 2019; Eitze *et al*., 2024).

Vaccination is an individual health decision (Dubé *et al*., 2013; Schmid *et al*., 2017) shaped by various contextual factors, including historical, sociocultural, environmental, healthcare system, economic, and political influences; individual and group dynamics; and vaccine-related issues (Plotkin, 2005). Additionally, the diverse sources of vaccine information play a crucial role in the decision-making process (Kestenbaum and Feemster, 2015).

In today’s world, the internet has become a major source of health information (Dubé *et al*., 2013). The internet and social media are increasingly used to communicate, access information, and make decisions regarding vaccination. Several studies have shown that individuals who report the internet as their main source of vaccine information tend to exhibit more vaccine hesitancy (Fabry *et al*., 2011; Betsch and Sachse, 2012; Wheeler and Buttenheim, 2013; Dubé *et al*., 2021). Furthermore, studies examining vaccine-related content on websites and social media platforms have shown that the quality of information varies widely, with significant amounts of misinformation present (Dubé *et al*., 2015).

Since vaccine hesitancy was identified as a global challenge, scientists and professionals have made several efforts to quantitatively evaluate why individuals avoid vaccination despite the availability of safe and effective vaccines. In 2018, Betsch and colleagues developed a model to measure five psychological antecedents of vaccination behavior, building on previous vaccine hesitancy and confidence models (Betsch *et al*., 2015; MacDonald, 2015; Thomson *et al*., 2016). This model, known as the *5C model (Confidence, Complacency, Constraints, Calculation, Collective Responsibility)*, evaluates key psychological dimensions influencing vaccination behavior. ‘*Confidence*’ refers to trust in vaccine effectiveness, safety, and necessity in healthcare providers and the healthcare system. ‘*Complacency*’ is the lack of perceived risk from vaccine-preventable diseases. ‘*Constraints*’ assess barriers to vaccination, such as availability, accessibility, and affordability. ‘*Calculation*’ examines individuals’ motivation to weigh the pros and cons of vaccination, while ‘*Collective Responsibility*’ reflects a social or communal sense of duty aimed at achieving herd immunity (Betsch *et al*., 2019; Betsch *et al*., 2020; Aggarwal *et al*., 2024; Eitze *et al*., 2024).

As vaccine hesitancy grows, we are witnessing significant public health consequences. With the decline in vaccination rates, outbreaks of vaccine-preventable diseases are resurging (Porter and Goldfarb, 2019). To improve vaccine uptake, it is essential to understand the factors and psychological precursors influencing individuals’ vaccine attitudes and vaccination behavior.

Research on the psychological precursors affecting vaccination behavior primarily focused on parents, healthcare workers, and vulnerable groups’ attitudes and behaviors regarding COVID-19 and childhood vaccinations. However, there is a recognized need for studies addressing general vaccine hesitancy among adults in Turkey, as these psychological precursors have not yet been explored in the context of vaccination-related attitudes and behaviors. To address this gap and understand regional differences at the local level in Ankara, this study targets adults and general vaccine hesitancy at a regional level.

This study aims to evaluate the psychological precursors and factors that shape vaccine attitudes and behaviors among adults who visit family health centers, addressing a critical gap in the literature on general vaccine hesitancy in Turkey.

## Material and methods

The reporting of this study follows the Strengthening the Reporting of Observational Studies in Epidemiology (STROBE) statement (Von Elm *et al*., 2007).

The study population consists of individuals aged 18 and over who visited some Family Health Centers in Ankara, the capital of Turkey.

The sample size was calculated using the EpiInfo software. Based on a 95% confidence interval, *α* = 0.05, a margin of error (*d*) = 0.025, design effect of 1.0, and anticipated frequency of 50%, the calculated sample size was 1504. A margin of error was selected to balance precision and feasibility of data collection, with smaller values improving estimate accuracy, particularly for subgroup analyses (Althubaiti, 2023), and as no cluster sampling was applied, the design effect was set to 1.0 consistent with survey methodology literature (Lohr, 2021). With an anticipated 10% data loss, the study aimed to survey 1650 individuals, ultimately reaching 1712 participants. Participants were recruited through a **convenience sampling method** among adults attending several Family Health Centers in Ankara. Individuals aged 18 years or older who were able to understand Turkish and voluntarily agreed to participate were included in the study. Those who had serious communication difficulties were excluded. During the data collection process, a total of **1768 individuals** were approached, and **56 declined to participate,** resulting in **1712 valid responses** included in the final analysis.

The research form, administered through face-to-face interviews, included questions about socio-demographic characteristics, individuals’ attitudes and behaviors toward vaccination, and internet usage. The Turkish version of the ‘Psychological Antecedents of Vaccination (5C) Scale’ was included (Demir *et al*., 2022). The data collection tool was a structured questionnaire developed by the researchers based on the literature. It consisted of four sections: 12 sociodemographic questions, the 15-item ‘Psychological Antecedents of Vaccination (5C) Scale,’ 8 questions evaluating vaccination attitudes and behaviors, and 7 items on internet use.

The Turkish short version of the 5C Scale, validated by Demir *et al*., was used in this study (Demir *et al*., 2022). Each psychological dimension (confidence, complacency, constraints, calculation, and collective responsibility) consisted of three items scored on a 7-point Likert scale (1 = strongly disagree, 7 = strongly agree). Dimension scores were calculated by summing the three corresponding items for each domain.

No pilot test was conducted prior to data collection; however, the questionnaire was reviewed by field experts to ensure content validity.

The independent variables, socio-demographic characteristics, included age, gender, presence of children, education level, employment status, income status, presence of chronic diseases, medication use, and health status. Questions about internet use and active social media use were also included. Household income was assessed subjectively using participants’ perceived income–expense balance. Participants were asked, ‘What is your household’s approximate total monthly income?’ with five response options: ‘1’ much less than expenses, ‘2’ slightly less, ‘3’ equal, ‘4’ slightly more, and ‘5’ much more than expenses. This approach reduces response bias by allowing participants to report their perceived economic situation.

The dependent variables were individuals’ self-reported vaccination behaviors and their general intentions to get vaccinated. Vaccination behavior regarding flu and COVID-19 vaccines was questioned. Individuals were asked, ‘In necessary situations (such as epidemic risk exposure or routine prevention), would you get vaccinated without hesitation?’ Responses were evaluated as ‘Yes/No’ and classified dichotomously.

Descriptive statistics for categorical variables are presented in tabular format, showing counts and percentages. The normality of continuous variables was evaluated using visual methods (histograms and probability plots) and analytical methods (Kolmogorov-Smirnov/Shapiro-Wilk tests). Data related to the dimensions of the 5C model, which did not follow a normal distribution, were presented using medians and minimum-maximum values. In bivariate analyses, the appropriate statistical tests, such as chi-square, Fisher’s exact test, and Mann-Whitney U tests, were used as needed.

Vaccination intention was assessed, and then the determinants of vaccination intention, including the 5C psychological antecedents (*confidence, complacency, constraints, calculation, collective responsibility*), were evaluated using logistic regression analysis. Factors with *p-values < 0.05* from bivariate analyses were included in the multivariate logistic regression model, considering potential causal relationships. To ensure the robustness of the model, multicollinearity among independent variables was examined, and only non-collinear variables were retained. However, since some variables lost their statistical significance in the multivariate model, only the significant predictors were presented in the results table (Table 5). Age was included as a continuous variable. The ‘*Backward LR*’ method was used to obtain the final model. No missing data were observed; therefore, all analyses were conducted on complete cases. All analyses were conducted using SPSS 23.0 (IBM Corp., Armonk, NY, USA) statistical software. The statistical significance level was accepted as *p* < 0.05 for this study. The study was conducted under the Helsinki Declaration. The Gazi University Ethics Committee reviewed and approved it on 13.02.2024, with decision number 2024–249. Written informed consent was obtained from all participants prior to data collection.

## Results

A total of 1712 participants were included in the study. The mean age of the participants was 46.7 ± 18.1 years. More than half of the participants were female (57.9%) and had at least a high school education (66.3%). Of the participants, 760 (44%) responded ‘No’ to the question about getting vaccinated without hesitation in necessary situations, while 952 (56%) indicated they would get vaccinated without hesitation in such situations. Vaccination intention was higher among males, individuals aged 65 and over, university graduates, those whose income exceeds their expenses, and those who use medications. There was no statistically significant difference in vaccination intention based on working status, presence of chronic diseases, family history of chronic diseases, or perceived health status (Table 1).

**Table 1.** Assessment of vaccination intention based on certain descriptive characteristics of the participants

		Vaccination intention		
*n* = 1712	*n* (%)*	Yes (%)** *n* = 952	No (%)** *n* = 760	p ***
***Gender***				
Woman	991 (57.9)	531 (53.6)	460 (46.4)	***p*** **= 0.048**
Male	721 (42.1)	421 (58.4)	300 (41.6)	
***Age group***				
18–39^(a)^	648 (37.8)	352 (54.3)	296 (45.7)	***p* < 0.001** (a–c)(b–c)
40–64^(b)^	729 (42.6)	377 (51.7)	352 (48.3)	
65 and over ^(c)^	335 (19.6)	223 (66.6)	112 (33.4)	
***Child existence***				
Yes	1292 (75.5)	720 (55.7)	572 (44.3)	*p* = 0.861**
No	420 (24.5)	232 (55.2)	188 (44.8)	
***Educational status***				
Not graduated from any school ^(a)^	61 (3.6)	38 (62.3)	23 (37.7)	***p* = 0.012** (c–d)
Primary/elementary school^(b)^	516 (30.1)	292 (56.6)	224 (43.4)	
High school ^(c)^	486 (28.4)	241 (49.6)	245 (50.4)	
University and above^(d)^	649 (37.9)	381 (58.7)	268 (41.3)	
***Working status***				
Working	649 (37.9)	605 (56.9)	458 (43.1)	*p* = 0.164
Not working	1063 (62.1)	347 (53.5)	302 (46.5)	
***Income status***				
Income less than expenses^(a)^	616 (36.0)	318 (51.6)	298 (48.4)	***p* = 0.003** (a–c)
Income equals expense^(b)^	735 (42.9)	407 (55.4)	328 (44.6)	
Income exceeds expenses^(c)^	361 (21.1)	227 (62.9)	134 (37.1)	
***Presence of chronic disease***				
Yes	796 (46.5)	462 (58.0)	334 (42.0)	*p* = 0.059
No	916 (53.5)	490 (53.5)	426 (46.5)	
***Medications usage status***				
Yes	829 (48.4)	487 (58.7)	342 (41.3)	***p*** **= 0.011**
No	883 (51.6)	465 (52.7)	418 (47.3)	
***Family history of chronic disease***				
Yes	1020 (59.6)	550 (53.9)	470 (46.1)	*p* = 0.088
No	692 (40.4)	402 (58.1)	290 (41.9)	
***Perceived health status***				
Very good/good	914 (53.4)	82 (5 7.7)	60 (42.3)	*p* = 0.170
Middle	656 (38.3)	346 (52.7)	310 (47.3)	
Very bad/bad	142 (8.3)	524 (57.3)	390 (42.7)	

*Column Percentage **Row Percentage, ***Pearson Chi-Square Test.

Post Hoc analyses were evaluated with Bonferroni correction.

Bold values indicate statistically significant associations (*p* < 0.05).

Vaccination intention was higher among those who received the COVID-19 vaccine, those who had received the flu vaccine at least once, those who did not experience fear of injections, and those who managed to convince a relative to get vaccinated. No statistically significant difference was found regarding difficulties accessing vaccination services (Table 2).

**Table 2.** Assessment of vaccination intention based on participants’ attitudes and behaviors toward vaccination

		Vaccination intention		
*n = 1712*	=""> *n* (%)*	Yes (%)** *n* = 952	No (%)** *n* = 760	p ***
***Covid-19-vaccined***				
Yes	1570 (91.7)	924 (58.9)	646 (41.1)	**< 0.001**
No	142 (8.3)	28 (19.7)	114 (80.3)	
***Got a flu vaccine at least once***				
Yes^(a)^	542 (31.7)	364 (67.2)	178 (32.8)	**< 0.001** (a–b)
No^(b)^	1085 (63.3)	536 (49.4)	549 (50.6)	
I don’t remember ^(c)^	85 (5.0)	52 (61.2)	33 (38.8)	
***Fear of injection***				
Yes	328 (19.2)	163 (49.7)	165 (50.3)	**0.017**
No	1384 (80.8)	789 (57.0)	595 (43.0)	
***Convincing a relative who does not want to get vaccinated***				
Yes	733(42.8)	581 (79.3)	152 (20.7)	**< 0.001**
No	976(57.2)	371 (37.9)	608 (62.1)	
***Experiencing difficulty in accessing vaccination services***				
I didn’t experience any difficulty	1558 (91.0)	856 (54.9)	702 (45.1)	0.078
I experienced difficulty	154 (9.0)	96 (62.3)	58 (37.7)	

*Column Percentage **Row Percentage, ***Pearson Chi-Square Test.

Post Hoc analyses were evaluated with Bonferroni correction.

Bold values indicate statistically significant associations (*p* < 0.05).

Among those who do not use the internet, vaccination intention was higher in those who found internet sources useful for obtaining vaccination information. There was no statistically significant difference in vaccination intention regarding the frequency of internet use and the frequency of obtaining information about vaccines (Table 3).

**Table 3.** Assessment of vaccination intention based on internet and social media usage among participants

		Vaccination intention		
	*n* (%)*	Yes (%)** *n = 952*	No (%)** *n = 760*	p ***
***Being an internet user (n = 1712)***				
Yes	1340 (78.3)	712 (53.1)	628 (46.9)	**< 0.001**
No	372 (21.7)	240 (64.5)	132 (35.5)	
***Frequency of internet use (n = 1340)***				
Less than 1 hour	267 (19.9)	128 (47.9)	139 (52.1)	0.154
1–3 hours	540 (40.3)	291 (53.9)	249 (46.1)	
Over 3 hours	533 (39.8)	293 (55.0)	240 (45.0)	
***Using the internet to access vaccine*** ***information (n = 1712)***				
Yes	993 (58.0)	524 (52.8)	469 (47.2)	**0.005**
No	719 (42.0)	428 (59.5)	291 (40.5)	
***Frequency of the internet use for accessing*** ***vaccine information (n = 993)***				
Less than once a month	662 (66.7)	361 (54.4)	301 (45.5)	0.262
Monthly	199 (20.0)	96 (48.2)	103 (51.8)	
Once a week or more often	132 (13.3)	67 (50.8)	65 (49.2)	
***Finding the internet helpful in reaching*** ***vaccination information (n = 993)***				
Not useful/not useful at all^(a)^	77 (7.8)	26 (33.8)	51 (66.2)	**< 0.001** (a–c)(b–c)
I am undecided^(b)^	321 (32.3)	160 (49.8)	161 (50.2)	
Helpful/very helpful ^(c)^	595 (59.9)	338 (56.8)	257 (43.2)	
***Active social media use (n = 1712)***				
Yes	1205 (70.4)	634 (52.6)	571 (47.4)	**< 0.001**
No	507 (29.6)	318 (62.7)	189 (37.3)	
***The most common source is “health professionals”*** ***when accessing vaccine information***				
Yes	734 (42.9)	430 (58.6)	304 (41.4)	**0.032**
No	978 (57.1)	522 (53.4)	456 (46.6)	
***The most common source is “internet”*** ***when accessing vaccine information***				
Yes	541 (31.6)	268 (49.5)	273 (50.5)	**0.001**
No	1171 (68.4)	684 (58.4)	487 (41.6)	

*Column Percentage **Row Percentage, ***Pearson Chi-Square Test.

Post Hoc analyses were evaluated with Bonferroni correction.

Bold values indicate statistically significant associations (*p* < 0.05).

Vaccination attitudes and behaviors among the participants were evaluated based on the five psychological antecedents of vaccination. Individuals who would get vaccinated without hesitation in necessary situations had statistically higher *confidence* and *collective responsibility* score medians. In contrast, their *complacency* and risk *calculation* score medians were lower *(p* > 0.001) (Table 4).

**Table 4.** Assessment of total scores of the 5C psychological antecedents scale sub-dimensions and vaccination intention

(*n* = 1712)	Median (Min-Max)	Vaccination intention		p*
		Yes Median (Min-Max)	No Median (Min-Max)	
*Confidence*	17.00 (3.00–21.00)	18.00 (3.00–21.00)	13.00 (3.00–21.00)	***p*** **< 0.001**
*Complacency*	9.00 (3.00–21.00)	9.00 (3.00–21.00)	11.00 (3.00–21.00)	***p*** **< 0.001**
*Constraints*	5.00 (3.00–21.00)	5.00 (3.00–21.00)	6.00 (3.00–21.00)	*p* = 0.199
*Risk calculation*	20.00 (3.00–21.00)	19.00 (3.00–21.00)	21.00 (3.00–21.00)	***p*** **< 0.001**
*Collective responsibility*	17.00 (3.00–21.00)	19.00 (3.00–21.00)	16.00 (3.00–21.00)	***p*** **< 0.001**

*: Mann-Whitney *U* test.

Bold values indicate statistically significant associations (*p* < 0.05).

Individuals who had received the flu vaccine at any time had higher median scores for ***confidence*** (*p* < 0.001) and ***collective responsibility*** (*p* < 0.001) compared to those who had not, while their median scores for ***complacency*** (*p* < 0.001) were lower. (Individuals who did not remember whether they had ever received the flu vaccine were not included in the analysis) (Table 4).

The factors affecting vaccination intention were assessed in the multivariate logistic regression analysis. The likelihood of getting vaccinated without hesitation in necessary situations was higher among those whose income exceeds their expenses (OR: 1.532, 95%CI = 1.107–2.119), among those who received the COVID-19 vaccine (OR: 2.362, 95% CI = 1.429–3.906), and among those who do not actively use social media (OR: 1.417, 95%CI = 1.096–1.833). Additionally, higher **confidence** scores (OR = **1.268**, 95% CI = 1.231–1.306) and **collective responsibility** scores (OR = **1.083**, 95% CI = 1.046–1.122) were positively associated with vaccination intention, while **higher calculation** scores were negatively associated (OR = **0.932**, 95% CI = 0.899–0.965) (Table 5).

**Table 5.** Evaluation of factors affecting vaccination intention using multivariate logistic regression analysis

Parameters (*n* = 1712)	OR (odds ratio)	Confidence interval (%95CI)	*p*-Value
*Household income*			
*Income is less than expenses*	*Ref*	–	–
*Income equals expense*	1.129	0.874–1.458	0.354
*Income is more than expenses*	1.532	1.107–2.119	**0.010**
*COVID 19-vaccinated*	2.362	1.429–3.906	**0.001**
*Not actively using social media*	1.417	1.096–1.833	**0.008**
*Confidence*	1.268	1.231–1.306	**< 0.001**
*Risk calculation*	0.932	0.899–0.965	**< 0.001**
*Collective responsibility*	1.083	1.046–1.122	**< 0.001**

*p* < 0.001 (Model)

Nagelkerke R square : 0.376

Hosmer and Lemeshow Test: 0.730

Bold values indicate statistically significant associations (*p* < 0.05).

## Discussion

In this study, more than half of the participants indicated they would not hesitate to get vaccinated when necessary. In a study conducted among U.S. adults, half of the participants showed no hesitancy toward COVID-19 vaccines or vaccines (Nguyen *et al*., 2024). While most people (55–75%) had a positive attitude toward vaccines, the exact prevalence of indecision remains unclear (Kader, 2019).

Vaccination intent was higher among men, individuals aged 65 and older, university graduates (compared to high school graduates), those whose income exceeded their expenses, and those taking medications. No statistically significant difference was found regarding vaccination intent related to employment status, the presence of chronic illness, family history of chronic disease, or perceived health status. However, in the multivariate analysis, the only sociodemographic feature that stood out was having an income higher than expenses.

The higher vaccination intent among older individuals than younger adults may stem from concerns that their natural immunity might not sufficiently protect them from infectious diseases or from being less likely to encounter misinformation online.

In a large-scale study examining vaccination attitudes in 67 countries, socioeconomic status and income were found to influence vaccination intent, which is consistent with the findings of this study. Similarly, individuals from lower-income groups had less favorable attitudes toward vaccines than higher-income groups. People aged 65 and older were more likely to report that vaccines were effective. Holding a master’s or doctoral degree – considered the highest level of education – was not associated with more positive views on vaccine importance and effectiveness when compared to those with no education. (Larson *et al*., 2016)

The study found no difference in vaccination intent between university and primary school graduates. However, individuals with university or higher education levels had greater vaccination intent compared to high school graduates. It is believed that those with lower education levels tend to accept health professionals’ vaccine recommendations without question; in contrast, university graduates and those with higher education are less indecisive because they base their vaccination decisions on reliable information. Individuals with intermediate education levels may research vaccines but struggle to distinguish accurate information from misinformation.

Existing research shows that in studies conducted in the United Arab Emirates and Saudi Arabia, participants with higher education levels were more skeptical of vaccines (Alsubaie *et al*., 2019; Nour *et al*., 2022). A broad research found that higher education was a potential barrier to vaccination in China, Lebanon, Israel, Bangladesh, and the United States. In contrast, studies conducted in the Netherlands, Nigeria, Pakistan, and Greece showed that higher education was associated with greater support for vaccination (*WHO Report of the Sage Working Group on Vaccine Hesitancy*, 2014).

In Greece, vaccine hesitancy was associated with certain sociodemographic characteristics such as age, place of residence, education level, and individual annual income (Gialama *et al*., 2024). Other studies investigating vaccine hesitancy in various countries confirmed the relationship between age, education level, and hesitancy (Mohd Azizi *et al*., 2017; Sasse *et al*., 2024).

In a study among Asian Americans living in New Jersey, no statistically significant relationship was found between vaccine hesitancy and education level, employment status, employment type, or household income. However, men were found to be less likely than women to exhibit vaccine hesitancy (Rana *et al*., 2024).

In a study representative of the French adult population, similar to the current study, the male gender was associated with the ‘non-hesitant’ group. These findings confirm that men tend to have more positive attitudes toward vaccines, while women are more likely to be hesitant (Khoury *et al*., 2024).

In the multivariate model, having an income higher than expenses emerged as an essential factor. Previous research on per capita income has reported higher vaccine hesitancy among lower-income groups than their higher-income counterparts (Hughes *et al*., 2021; Guo *et al*., 2022; Sasse *et al*., 2024).

Individuals with higher socioeconomic status may have access to more accurate health information or know how to access reliable sources better. Even if vaccines are free, indirect costs like taking time off work or traveling to a vaccination site could deter some from getting vaccinated. Income inequality likely affects people’s health-related attitudes and decision-making processes.

Vaccination intent was higher among those who had received the COVID-19 vaccine, those who had at least one flu shot, those not afraid of injections, and those who had persuaded a hesitant relative to get vaccinated.

In the multivariate model, receiving the COVID-19 vaccine emerged as the most significant determinant of general vaccination intent. This aligns with findings from Aggarwal *et al*., highlighting that vaccination behaviors during the COVID-19 pandemic remain crucial for understanding vaccine hesitancy and its behavioral determinants (Aggarwal *et al*., 2024).

In a study by Yang *et al*., which evaluated the relationship between vaccine hesitancy and vaccination behavior, individuals with vaccine hesitancy had a higher risk of not getting vaccinated than non-hesitant individuals (Yang *et al*., 2024).

Approximately 8 out of every 10 participants reported using the internet, and 6 out of 10 indicated they used it to access information about vaccines. The most frequently consulted sources of vaccine information were health professionals and the internet. Vaccination intent was higher among those who did not use the internet to find vaccine information, those who did not actively use social media, and those who primarily relied on health professionals for vaccine-related information.

In a study by Alzahrani *et al*., similar to this study, the most common sources of vaccine information were reported to be doctors, the internet, and social media. The study found a significant difference in vaccine hesitancy rates depending on the information sources (Alzahrani and Alghamdi, 2023). Skafle *et al*. synthesized the results of 19 studies measuring the impact of misinformation on social media and concluded that social media content negatively influenced vaccine hesitancy and uptake (Skafle *et al*., 2022). Other studies have also found that individuals who rely on social media for vaccine information are more likely to be hesitant (Dambadarjaa *et al*., 2021; Horiuchi *et al*., 2021; Al-Amer *et al*., 2022; Brackstone *et al*., 2022; Alzahrani and Alghamdi, 2023; Aleksandric *et al*., 2024).

In a study by Huang *et al*., vaccine hesitancy was lower among those who received clinical recommendations, consistent with this study’s findings (Huang *et al*., 2022).

In a study by Nikoloski *et al*., individuals who were hesitant about vaccination had less trust in healthcare workers and relied more on social media for COVID-19-related information. Those who received information from healthcare providers were more likely to get vaccinated; however, hesitant individuals relied more on private healthcare providers, social media, and the internet for information (Nikoloski *et al*., 2023).

Unlike traditional media, social media allows individuals to rapidly create and share content globally without editorial oversight, which can lead to the rapid spread of misinformation (Puri *et al*., 2020). The spread of misinformation undermines trust in science and contributes to increasing vaccine hesitancy (Huang *et al*., 2022; Kosiyaporn *et al*., 2023; Ugarte and Young, 2023). Many people lack adequate health and digital literacy when searching for reliable health information, and social and economic inequalities exacerbate this (Kbaier *et al*., 2024). To reduce the impact of social media and the internet on vaccine intent, improving individuals’ ability to find, understand, evaluate, and apply vaccine-related information online – known as digital vaccine literacy – is crucial.

When evaluating the psychological factors affecting vaccine attitudes and behaviors, it was found that those who would not hesitate to get vaccinated, those who had received the COVID-19 vaccine, and those who had been vaccinated for the flu had higher *confidence* and *collective responsibility* scores, while their *complacency* scores were lower. Interestingly, general vaccination intent was associated with lower *calculation* scores, while COVID-19 vaccine recipients had lower *constraint* scores.

Van de Berg *et al*.’s assessment of the 5C psychological factors related to COVID-19 vaccination suggested that interventions to increase COVID-19 vaccination coverage should address *confidence* and *complacency* (van de Berg *et al*., 2024). In studies conducted in other countries, *collective responsibility* (Alenezi *et al*., 2022) and *calculation* (Wagner *et al*., 2022) were found to influence vaccination and intent, respectively. This highlights the importance of evaluating vaccination determinants specific to each region, as different psychological factors may have varying impacts across societies. While *confidenc*e and *complacency* commonly influence vaccine attitudes and behaviors, the effects of *collective responsibility* and *calculation* may differ across communities.

In a study by Sanftenberg *et al*., no significant relationship was found between the psychological factors of vaccination and seasonal flu or pneumococcal vaccination. However, *confidence*, *complacency*, and *constraint*s were influential factors for COVID-19 vaccination (Sanftenberg *et al*., 2024). Studies suggest that different psychological factors may play a role in each vaccine. Unlike Sanftenberg *et al*., this study found that the psychological factors influencing general vaccination intent were similar to those influencing flu vaccination, except that COVID-19 vaccination was significantly affected by the *constraints* factor. These *constraints* could be related to the impact of the pandemic on healthcare services and the initial lack of an available vaccine.

In the multivariate analysis evaluating factors influencing vaccination intent, psychological factors – rather than demographic variables – were significant predictors of vaccine intent. Increases in *confidence* and *collective responsibility* were associated with higher vaccination intent, while higher *calculation* scores were linked to lower intent. Although *complacency* was statistically significant in the binary comparison, it did not retain significance in the multivariate model and was therefore not included in the final reported results. Receiving the COVID-19 vaccine, having a high socioeconomic status, and not actively using social media were other significant predictors of non-hesitant vaccination.

The existing literature, like this study, shows that individuals’ trust in vaccines and their sense of responsibility to prevent the spread of disease meaningfully increase vaccination intent. The 5C model supports that psychological factors such as ‘Confidence’ and ‘Collective Responsibility’ are key to reducing vaccine hesitancy. In these studies, participants had confidence in the safety and efficacy of vaccines and were motivated by a sense of social responsibility and collective action to reduce infection within their communities (Paterson *et al*., 2016; Chaturvedi *et al*., 2019; Dhalaria *et al*., 2022; Limbu and Gautam, 2023; Aggarwal *et al*., 2024).

*Confidence* is a prominent psychological factor influencing vaccine attitudes and behaviors, appearing in nearly every related study. Interventions designed to enhance trust can foster positive perceptions of vaccines by boosting confidence in their safety and effectiveness (Betsch *et al*., 2019; van de Berg *et al*., 2024).

A study conducted in Switzerland by Wagner and colleagues found that increased deliberation reduced vaccination intention (Wagner *et al*., 2022). Both studies found that when adults carefully weighed the benefits and risks of vaccines, their intention to vaccinate decreased. How individuals assess these benefits and risks and which information sources they rely on is critical. Commonly used information sources, like the internet and social media, tend to emphasize risks and side effects. Furthermore, it is known that the quality of information on these platforms is inconsistent, with inaccurate or harmful content often being more prominent (Dubé *et al*., 2013). This may lead to increased deliberation, which could decrease vaccination intention.

This study has some limitations. Since participation was voluntary, the sample’s representativeness is considered limited. Moreover, as adults in the middle-age group are known to use Family Health Centers less frequently than younger or older adults, the findings may not fully reflect the vaccination attitudes of the general adult population. Additionally, conducting research among individuals who visited a primary healthcare facility where vaccinations are available may indicate that these individuals have greater trust in basic health services, potentially leading to higher vaccination intention than the general population.

## Conclusions

Receiving the COVID-19 vaccine, having a higher socioeconomic status, not actively using social media, trust, collective responsibility, and psychological factors such as ‘*calculation*’ are key determinants of getting vaccinated without hesitation. To improve vaccine acceptance, communication strategies should focus on building *confidence* and *collective responsibility*. It is also essential to investigate the risk factors that lead to vaccine hesitancy among individuals who actively use social media, and **strategies** should be implemented to address these. Furthermore, efforts should be made to enhance individuals’ digital vaccine literacy skills.

*Confidence* and *collective responsibility* scores were higher, while *complacency* scores were lower across all three cases: general vaccination intent, the COVID-19 vaccine, and the flu vaccine when assessing the psychological precursors of vaccination attitudes and behaviors. In vaccination campaigns and communication strategies ***, collective responsibility*** should be emphasized alongside ***confidence.*** The consequences of not getting vaccinated should be communicated directly and efficiently to prevent complacency.

In contrast, while the ‘*calculation*’ score was low in individuals with general vaccination intent, those who had received the COVID-19 vaccine had low ‘*constraints*’ scores. These differences should not be overlooked when preparing interventions to increase vaccine coverage. It’s essential to remember that the psychological precursors affecting general vaccination intent and specific vaccination behavior may vary.

To increase vaccination intent, efforts should prioritize building trust in vaccines and the vaccination process, highlighting the benefits of vaccination, and fostering a sense of social responsibility. Addressing the reasons behind people’s negative attitudes toward vaccination is key. Launching mass awareness campaigns on vaccine acceptance would be beneficial. Accurate information and messages could be disseminated through mass media and healthcare workers who are trained on this issue.

The findings of this study indicate that vaccination intent is strongly influenced not only by demographic factors but also by psychological antecedents such as trust and collective responsibility. This has important public health implications, suggesting that interventions to promote vaccination should go beyond informational campaigns and incorporate strategies that build trust and reinforce a sense of social responsibility.

Moreover, the association between lower vaccination intent and higher calculation tendencies or active social media use points to the need for more targeted efforts to combat misinformation and cognitive overload in digital environments. Public health policies should consider psychological drivers and develop more comprehensive approaches that address both emotional and cognitive aspects of vaccine hesitancy.

## Data Availability

The data that support the findings of this study are available from the corresponding author upon reasonable request.
